# Deletion of *REXO1L1* locus in a patient with malabsorption syndrome, growth retardation, and dysmorphic features: a novel recognizable microdeletion syndrome?

**DOI:** 10.1186/s12881-015-0164-3

**Published:** 2015-04-02

**Authors:** Maria Rosaria D’Apice, Antonio Novelli, Alessandra di Masi, Michela Biancolella, Antonio Antoccia, Francesca Gullotta, Norma Licata, Daniela Minella, Barbara Testa, Anna Maria Nardone, Giampiero Palmieri, Emma Calabrese, Livia Biancone, Caterina Tanzarella, Marina Frontali, Federica Sangiuolo, Giuseppe Novelli, Francesco Pallone

**Affiliations:** Fondazione Policlinico Tor Vergata, Rome, Italy; Mendel Institute, IRCCS Casa Sollievo della Sofferenza, San Giovanni Rotondo, Italy; Department of Biology, “Roma Tre” University, Rome, Italy; Department of Biomedicine and Prevention, Tor Vergata University of Rome, Rome, Italy; Department of Neuroscience, Psychiatry and Anaesthesiology, University of Messina, Messina, Italy; Pathological Anatomy Unit, University Tor Vergata, Rome, Italy; Department of Internal Medicine, Gastrointestinal Unit, Tor Vergata University of Rome, Rome, Italy; Institute of Translational Pharmacology, CNR Rome, Italy; San Pietro Fatebenefratelli Hospital, Rome, Italy

**Keywords:** 8q21.2 microdeletion, *REXO1L1* gene, aCGH, CNV, Facial dysmorphisms, Inflammation and apoptosis of gastrointestinal mucosa

## Abstract

**Background:**

Copy number variations (CNVs) can contribute to genetic variation among individuals and/or have a significant influence in causing diseases. Many studies consider new CNVs’ effects on protein family evolution giving rise to gene duplicates or losses. “Unsuccessful” duplicates that remain in the genome as pseudogenes often exhibit functional roles. So, changes in gene and pseudogene number may contribute to development or act as susceptibility alleles of diseases.

**Case presentation:**

We report a *de novo* heterozygous 271 Kb microdeletion at 8q21.2 region which includes the family of *REXO1L* genes and pseudogenes in a young man affected by global development delay, progeroid signs, and gastrointestinal anomalies. Molecular and cellular analysis showed that the *REXO1L1* gene hemizygosity in a patient’s fibroblasts induces genetic instability and increased apoptosis after treatment with different DNA damage-induced agents.

**Conclusions:**

The present results support the hypothesis that low copy gene number within *REXO1L1* cluster could play a significant role in this complex clinical and cellular phenotype.

## Background

Microarray-based comparative genomic hybridization (aCGH) is the current molecular technique used to diagnose submicroscopic deletions or duplications with higher resolution than classical cytogenetic banding in a single assay. It has applied to clinical diagnostics of patients with dysmorphic features, developmental delay, and/or idiopathic mental retardation and to delineate alterations that could be used to classify different subtypes of human tumours [1,2].

Moreover, the application of array CGH has led to the detection of large numbers of structural genomic rearrangements known as copy number variations (CNVs) in patients and in the normal population. CNVs can represent benign polymorphic variants, driving gene and genome evolution. The current challenge is the interpretation of the CNVs clinical significance in sporadic traits and in causing susceptibility to complex diseases [3,4]. In fact, the number of microdeletion and microduplication syndromes (MMSs) and the phenotypic consequences is continuously increasing [5]. Here, we describe a patient with malabsorption syndrome, growth retardation, dysmorphic features and dyspraxia associated with enhanced epithelial cells apoptosis in the gastrointestinal tract. Array-CGH analisis showed a heterozygous *de novo* microdeletion mapping in 8q21.2 band containing the *REXO1L1* gene and 3 *REXO1L2P* pseudogenes. We demonstrate that the observed chromosome deletion could be causative of the clinical and cellular phenotype observed in the patient.

## Case presentation

### Clinical report

The patient was born preterm by vaginal delivery, showing 2.900 Kg weight at birth. He underwent surgery to correct a cleft of the soft palate, while the incomplete spina bifida, diagnosed when he was a newborn, not required surgical treatment. At age 4, he had a diagnosis of dyspraxia, requiring regular Psichiatry Day Hospital admissions till 18 years old. At age 17, growth retardation and delayed puberty were diagnosed. An extensive paediatric work up revealed a short stature, mildly increased Body Mass Index (BMI), dyspraxia and osteoporosis (reduced age-related bone mass: T score −2.56, Z score = −2.31). At age 22, he referred to our gastrointestinal unit for chronic diarrhoea with weight loss not related with reduced food intake, and no responsiveness to anti-diarrhoeal drugs. At the time of admission, the patient appeared in poor conditions and older than his age. Physical examination revealed several dysmorphic features, including large palpebral fissures with long eyelashes, arched eyebrows, large ears, micrognathia, hypodontia, few and rare hair, together with cleft palate and velum pendulum bifidum. Routine blood chemistry detected reduced serum levels of total IgA (35 mg/dL; n.v. 70–400) and IgE (0 UI/ml; n.v. 20–100 UI/ml). A low grade hypoprotidemia (6.4 gr/dL) and hyperbilirubinemia (total 1.34 mg/dl, direct 0.39 mg/dL) were observed. The mean daily stools weight (2 determinations in 24 hours) was 1117 gr/24 hr, with steatorrhoea (8 gr/24 hr) and a positive occult faecal blood test.

**Figure 1:**
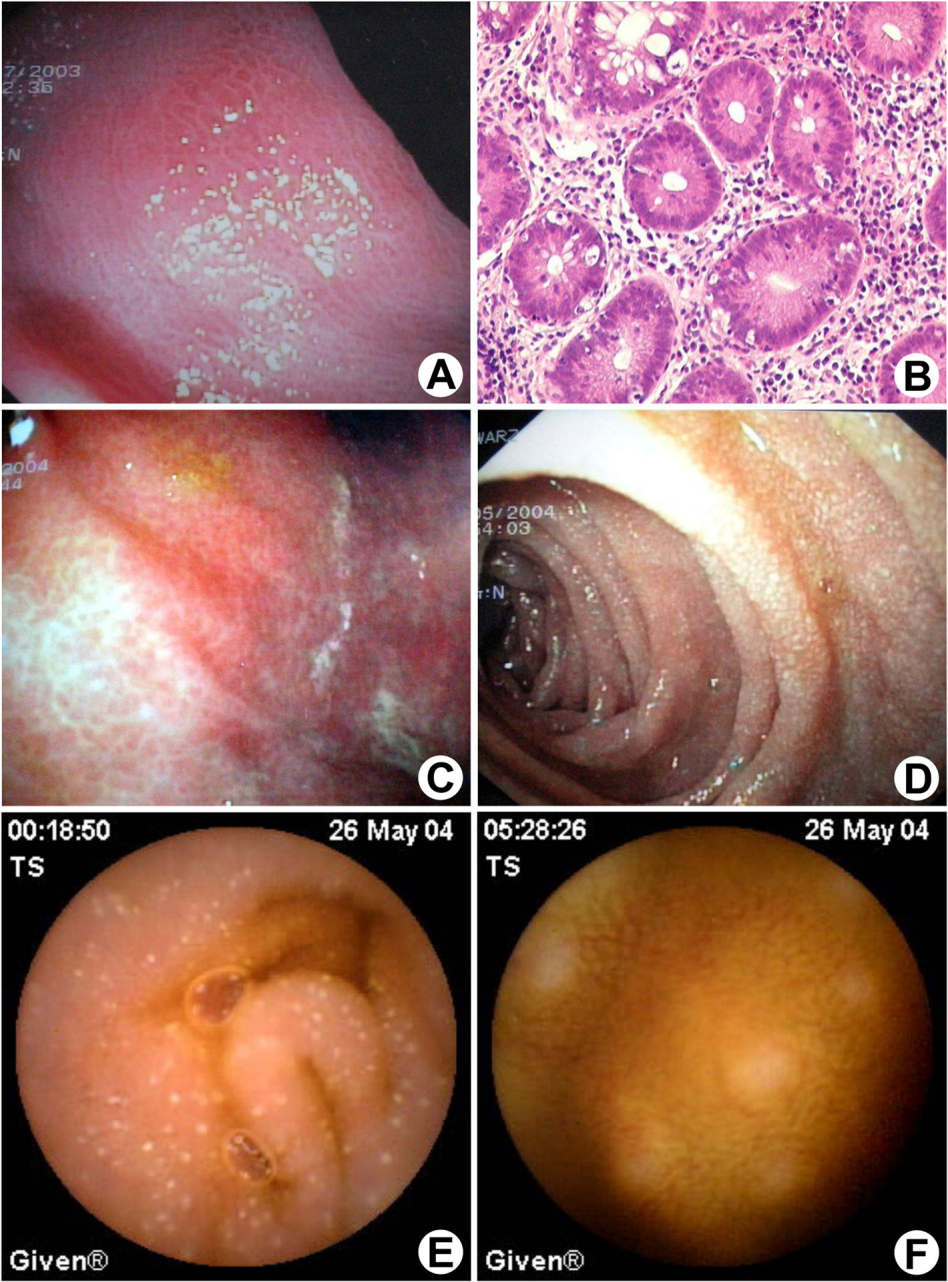
**Ileocolonoscopy and esophagogastroduodenoscopy. (A)** Presence of multiple areas of brownish “alligator skin” appearance of the intestinal mucosa, associated with disappearance of the vascular pattern and tubular aspect of the colon. **(B)** Histological analysis of a biopsy specimen taken from the distal ileum during ileocolonoscopy showed several apoptotic cells in along the epithelial cells lining the crypts (arrows). **(C)** EGDS of the gastric antrum showed multiple areas of brownish (“alligator skin”) appearance of the mucosa, in the absence of active erosions or ulcers. **(D)** The second portion of the duodenum showed multiple whitish spots compatible but not specific for lymphangiectasia. **(E)** SBCE showed the presence of multiple whitish spots in the second part of the duodenum (confirming findings at EGDS) extending to the proximal jejunum. **(F)** SBCE showed multiple subcentimetric nodular areas covered by normal mucosa in the jejunum and ileum, compatible with nodular lymphoid hyperplasia.

Esophagogastroduodenoscopy (EGDS) detected a normal macroscopical aspect of the Kerkring folds in the second portion of the duodenum, with multiple whitish spots compatible, but not specific, for lymphangiectasia [6]. However, focal areas with partial atrophy of the villi and an increased inflammatory infiltrate in the lamina propria were observed. Ileocolonoscopy showed multiple areas of brownish “alligator skin” appearance of the intestinal mucosa were observed, associated with disappearance of the vascular pattern and tubular aspect of the colon (Figure 1A). In the distal ileum, histological analysis showed an increased inflammatory infiltrate with occasional apoptotic bodies within the crypts (Figure 1B). Microscopic analysis of biopsy samples of colon detected an increased infiltration of plasmacells and eosinophils. Diffuse mucous depletion and apoptotic bodies within the crypts and at the basal portion of the glands were also observed. These findings were more relevant in the rectum, ascending and descending colon, when compared to the ileo-cecal valve. Mucosal atrophy was also observed. After treatment with probiotics and loperamide, partial and temporary reduction of the daily bowel movements, associated with no weight gain, have been observed.

Due to the persistence of diarrhoea, a gluten-free diet was started, followed by temporary resolution of this symptom (1 bowel movement every 2 days). In a successive reassessment of the disease, the patient appeared in discrete general conditions. A new EGDS showed a brownish mucosa covering the fundus, corpus and antrum, with no active erosions or ulcers (Figure 1C). The second portion of the duodenum showed multiple whitish spots, comparable to those reported in the previous EGDS (Figure 1D). Biopsy taken from the angulus, antrum, corpus and fundus showed a mild inflammatory infiltrate in the lamina propria, occasional lymphoid aggregates and limited aspects of foveolar hyperplasia. More importantly, focal apoptotic glandular necrosis was observed in the fundic mucosa. Histology of the second portion of the duodenum detected aspecific dysmorphism of the villi, mild increase of the inflammatory infiltrate in the lamina propria, focal mucous depletion and regenerative dysplasia.

In order to search for superficial small bowel lesions, a small bowel capsule endoscopy (SBCE) confirmed the findings detected by EGDS, including the presence of multiple and diffuse whitish spots extending from the second portion of the duodenum to the proximal jejunum (Figure 1E) [7]. SBCE also showed in the jejunum and ileum multiple subcentrimetric nodular areas covered by normal mucosa, compatible with nodular lymphoid hyperplasia (Figure 1F). Routine blood chemistry during hospitalization detected a mild hypocholesterolemia (HDL) (31 mg/dl; n.v. 35–60) and hypoprotidemia (6.2 g/dl; n.v. 6.6-8.7) with normal serum albumin (3.9 g/dl) and lower levels of β1 (0.33 g/dl; n.v. 0.4-0.9) and β2 globulins (0.25-0.7 g/dl; n.v. 0.25-0.7).

## Methods

Research carried out on patient’s fibroblasts was performed in compliance with the Helsinki Declaration (http://www.wma.net/en/30publications/10policies/b3/index.html) and received the approval of the ethics committee of Policlinico Tor Vergata (153/08).

### Cytogenetic and molecular studies

Comparative genomic hybridisation (CGH) analysis was performed on genomic DNA (gDNA) using the SpectralChip™2600 (Spectral Genomics Inc., Houston, TX). Test and normal reference DNA were processed according to manufacturers’ instructions. Slides were scanned on a GenePix 4000B scanner (Axon Instruments, Union City, CA). The acquired microarray images were analyzed by SpectralWare software (Spectral Genomics Inc.), which calculates hybridization ratio for each clone in the two experiments and generates a profile for each chromosome.

Fluorescence in situ hybridisation (FISH) analysis was performed using RP11-96G1 (hg18 chr:86,851,750-86,955,528), RP11-133G2 (hg18 chr8:86,558,072-86,717,112), RP11-179B4 (hg18 chr8:86,988,241-87,124,169) BAC clones and centromeric probe of chromosome 8 on metaphase spreads obtained from peripheral blood of our patient and his parents using standard procedures.

Gene copy number variation analysis was performed on gDNAs extracted by sixty healthy blood donors, proband and his parents’s blood samples using 2^-ΔΔCt^ method [8]. We used, as calibrator, the *REXO1L1* (FAM) gene cloned into pCMV6-XL6 (OriGene Technologies, Inc., Rockville, USA), and as reference gene the *RNaseP* (VIC) gene (Applied Biosystems, Foster City, CA), that it is known to be present in the human haploid genome in single copy. PCR was carried out using 10 ng of gDNA, 12.5 μl of Master mix (Applied Biosystems) and 1.25 μl of *REXO1L1* and *RNaseP* commercial probes together or separately. The thermal cycling conditions were: 2′ at 50°C, 10′ at 95°C and for 40 cycles 15″ at 95°C and 1′at 60°C. PCR was performed in a 96-well optical plate (ABgene) using the ABI7000 Real Time PCR System (Applied Biosystems). All DNA samples were amplified in triplicate. Two standard curves, with a known copy number of calibrator and reference gene, were prepared in duplicate. A no-template control (negative control) was also included in each assay.

Real-time efficiencies were calculated by means of standard calibration curves and the initial concentration of the sample was calculated by using the comparative ΔΔCt method as the gene copy number was given by the formula 2^−(ΔΔCt+/−SD)^, where ΔΔCt = (Ct *RNaseP* calibrator − Ct *REXO1L1* calibrator) − (Ct *RNaseP* sample − Ct *REXO1L1* sample). The Ct value was determined by using the instrument’s software and adjusted manually as necessary.

*REXO1L1* gene expression was performed on total RNA isolated from peripheral lymphocyte, fibroblasts, and cancer cell lines by TRIzol® method (Invitrogen Ltd, Paisley, UK). Given the monoexonic structure of *REXO1L1* transcript, we incubate 3 μg of RNA with 2 U of DNase I enzyme (Ambion) at 37°C for 30 min. Then, the RNA was reverse-transcribed to cDNA using the High-Capacity cDNA Archive Kit (Applied Biosystems). The *REXO1L1* gene was amplified using specific primers (Fw AGCTCAAGGAGAACGGCTACC, Rw TTGTGGCCGTCCTGGCTGTCC). Obtained amplicon was checked by sequencing analysis, to make sure that only the specific product was amplified.

Four micrograms of total RNA, isolated from patient’s fibroblasts, were analyzed using GEArray S Series Human Apoptosis and Cell Cycle Gene Array HS-603 (SuperArray Bioscience Corporation), containing 96 key apoptosis genes, 96 key cell cycle regulation genes, 75 stress & toxicity genes, negative controls (pUC18 DNA and blanks), and putative housekeeping genes (*β-actin*, *GAPDH*).

### Cellular analysis

Human fibroblast HFFF2 cell line and patient’s fibroblasts were cultured in DMEM-F12 medium supplemented with 15% foetal calf serum (FCS) and 1% L-glutamine. The HeLa and the HEK293 cell lines were growth in DMEM medium supplemented with 10% FCS. All culture media contains antibiotics. All the cell cultures are growth at 37°C under an atmosphere of 5% CO_2._

For the radiation treatment, cells in exponential phase of growth were exposed to X-rays delivered by a Gilardoni MGL 300/6-D apparatus (Gilardoni, Mandello Lario, Italy), operating at a dose rate of 0.53 Gy/min (250 kV, 6 mA, Cu filter).

Micronuclei (MN) induction on HFFF2 and patient’s fibroblasts was performed by treatment with either 4–8 J/m2 of UV-C, or with 2–5 μM Hydroxyurea, or 25, 50 and 100 μM t Butil-hydroxiperoxide or 0.25, 0.5 and 1 Gy of X-rays, and exposition with cytocalasin B (3 μg/ml) for 72 hrs. Cells were then fixed in situ by the gradual adding of methanol:acetic acid (3:1), slides were air-dried and stained with 3% Giemsa for 10 min. At least 500 binucleate cells (BNC) were scored for MN induction for each experimental point.

DNA double strand breaks (DSBs) repair measurements were performed after treatment with 40 Gy and repair incubation (0, 1, 2, 6, 24 hrs). Cells were harvested, embedded in agarose plugs (Low Melting Gel Type VII, Biorad) and lysed. PFGE was carried out with a Chef Mapper™ (Pulsed Field Electrophoresis System, Bio-Rad) in 0.8% Certified Molecular Biology Agarose (Bio-Rad) and 0.5 X TAE at 14°C. The run was performed first for 65 hrs at 1.5 V/cm using a 50–5000 sec switching time, then for 4 hrs at 6 V/cm using a 7–114 sec switch time block. Gels were stained with ethidium bromide and photographed with Fluor-S Imager (Bio-Rad) under UV transillumination. Densitometry was performed with Multi-Analyst software (Bio-Rad). The amount of DNA entering the gel was quantified.

Immunofluorescence analysis of γ-H2AX foci was performed on normal and patient’s fibroblasts after irradiation with 1 Gy of X-rays and fixation after 0.5, 6 and 24 hrs in 2% paraformaldehyde. Slides were incubated over night with 1 μg/ml γ-H2AX mouse monoclonal antibody (Upstate), and detected with an anti-mouse FITC-conjugated antibody (Vector). Images were captured using a Zeiss Axioplan 2 imaging epifluorescent microscope equipped with a charge-coupled device camera (CCD camera) and IAS2000 software. Quantitative analysis was carried out by counting foci in at least 50 cells/experiments.

Apoptosis assay was performed on cells harvested after 48 hrs from X-irradiation, washed with cold phosphate-buffered saline (PBS) and fixed with 70% ethanol. Fixed cells were treated with 20 μg/ml RNase and finally DNA was stained by addition of 50 μg/ml propidium iodide solution for 30 min at 37°C. To determine the DNA content of each sample, 10.000 cells were analysed, using a Galaxy Flow Cytometer (Dako). Percentages of cells in the different phases of cell cycle and cells with hypodiploid DNA content were established using the FloMax (Version 2.4b, Partec).

## Results

**Figure 2:**
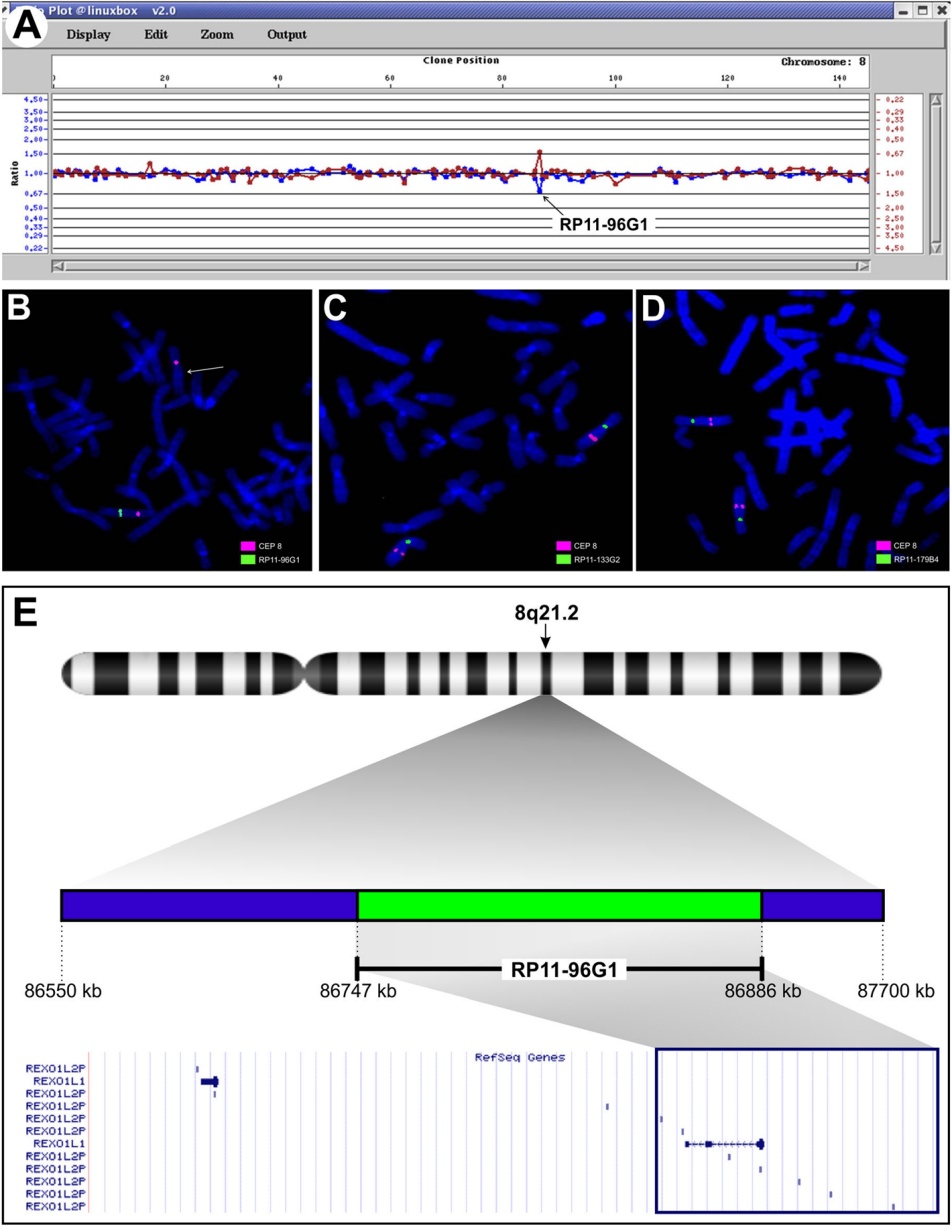
**Microdeletion characterization. (A)** For the array-CGH profile of chromosome 8, clones are ordered on the X axis from pter to qter according to physical mapping position. The Y axis (blue and red) mark the normalized hybridization Cy5:Cy3 and Cy3:Cy5 ratios of the two arrays. Black arrow showed microdeletion of a single clone (RP11-96G1) on 8q21.2 (ratio plot: 0.67 in both experiments). **(B)** Confirmation of array CGH results using FISH analysis with RP11-96G1 clone. White arrow shows the chromosome 8 containing the microdeletion. **(C–D)** FISH with RP11-133G2 and RP11-179B4, overlapping partially the deleted clone, showed that microdeletion spreads out along ~150 Kb. **(E)** The panel shows the deleted region in 8q21.2 cytogenetic band using the UCSC browser, in which the involved BAC clone and genes are displayed.

Analysis of the ratio profiles obtained by array-CGH showed a deletion of a single bacterial artificial chromosome (BAC) clone, RP11-96G1, mapping in 8q21.2 band (Figure 2A). To validate the results of CGH-array, a fluorescence *in situ* hybridization (FISH) analysis using the same clone was carried out. A single signal on one of the chromosomes 8 was detected in patient’s metaphase (Figure 2B). The deletion at 8q21 was also detected by qPCR. The patient’s genomic DNA contains half dose of region (data not shown). Both molecular techniques ruled out the presence of deletion in both parents, indicating its *de novo* origin. Then, the extension of the deleted region was determined using flanking clones (RP11-133G2 and RP11-179B4). FISH analysis with these probes showed one signal on both chromosomes 8, therefore microdeletion has been limited to RP11-96G1 clone, spanning for 104–271 Kb (arr[hg18] 8q21.2(86,717,112x2,86,851,750-86,955,528x1,86,988,241x2)) on 8q21.2 (Figure 2C-D). The deleted region contains the *REXO1L1* gene (NM_172239, REX1, RNA exonuclease 1 homolog (S. cerevisiae)-like 1, previously called *GOR*) and 3 *REXO1L2P* pseudogenes (REX1, RNA exonuclease 1 homolog (S. cerevisiae)-like 2) (Figure 2E). The analysis of copy number variation by qPCR revealed 8 copies of gene and pseudogenes in the proband’s DNA. All analyzed sixty healthy subjects displayed a variable number between 16–24 copies of *REXO1L1* gene and pseudogenes per diploid genome (data not shown).

**Figure 3:**
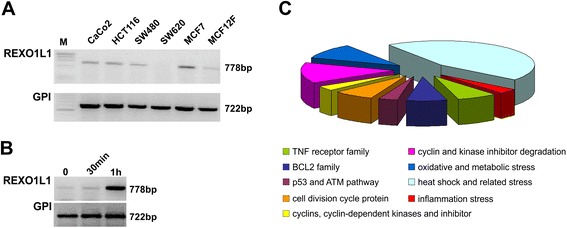
**Gene expression results. (A)** Relative expression of the *REXO1L1* gene measured in several cell lines by RT-PCR. CaCo-2, HCT116, SW480, and SW620 = human colon cancer cell lines, MCF-7 and MCF12F = human breast cancer cell lines, M = marker. **(B)** Relative expression of the *REXO1L1* gene measured by RT-PCR in HEK293T (Human Embryonic Kidney 293) cells after irradiation (5 Gy) as compared to untreated cells. **(C)** Differentially expressed genes identified by microarray analysis in proband’s fibroblasts are grouped according to the biological process in which are involved.

We examined the expression of the *REXO1L1* gene in several well-known cell lines by RT-PCR. *REXO1L1* gene is expressed in several mammary and gastrointestinal cancer cells (Figure 3A). Moreover, the *REXO1L1* gene produces an inducible mRNA, comparing the expression level in HEK293 cell line treated with 5 Gy of X-rays and harvested 30 min and 1 h later. A significant time-dependent up-regulation of *REXO1L1* gene was observed in irradiated cells (Figure 3B).

To investigate the functional effect of *REXO1L1* gene hemizygosity, we performed microarray analysis on the apoptosis and cell cycle regulation genes in patient’s fibroblasts. We used the GEArray S Series Human Apoptosis e Cell Cycle Gene Array HS-603, a filter array containing 96 key apoptosis genes, 96 key cell cycle regulation genes and 75 stress & toxicity genes. Considering only genes whose differential expression had a threshold > ±2, we identified a total of 29 differentially expressed genes (10.9%). A great number of these transcripts are heat shock genes (44.8%). The remaining group of altered genes are transcripts that take part in the regulation of protein turn-over (13%), cell cycle division (6.8%) and progression (3.4%), apoptosis (6.8%) (Figure 3C).

**Figure 4:**
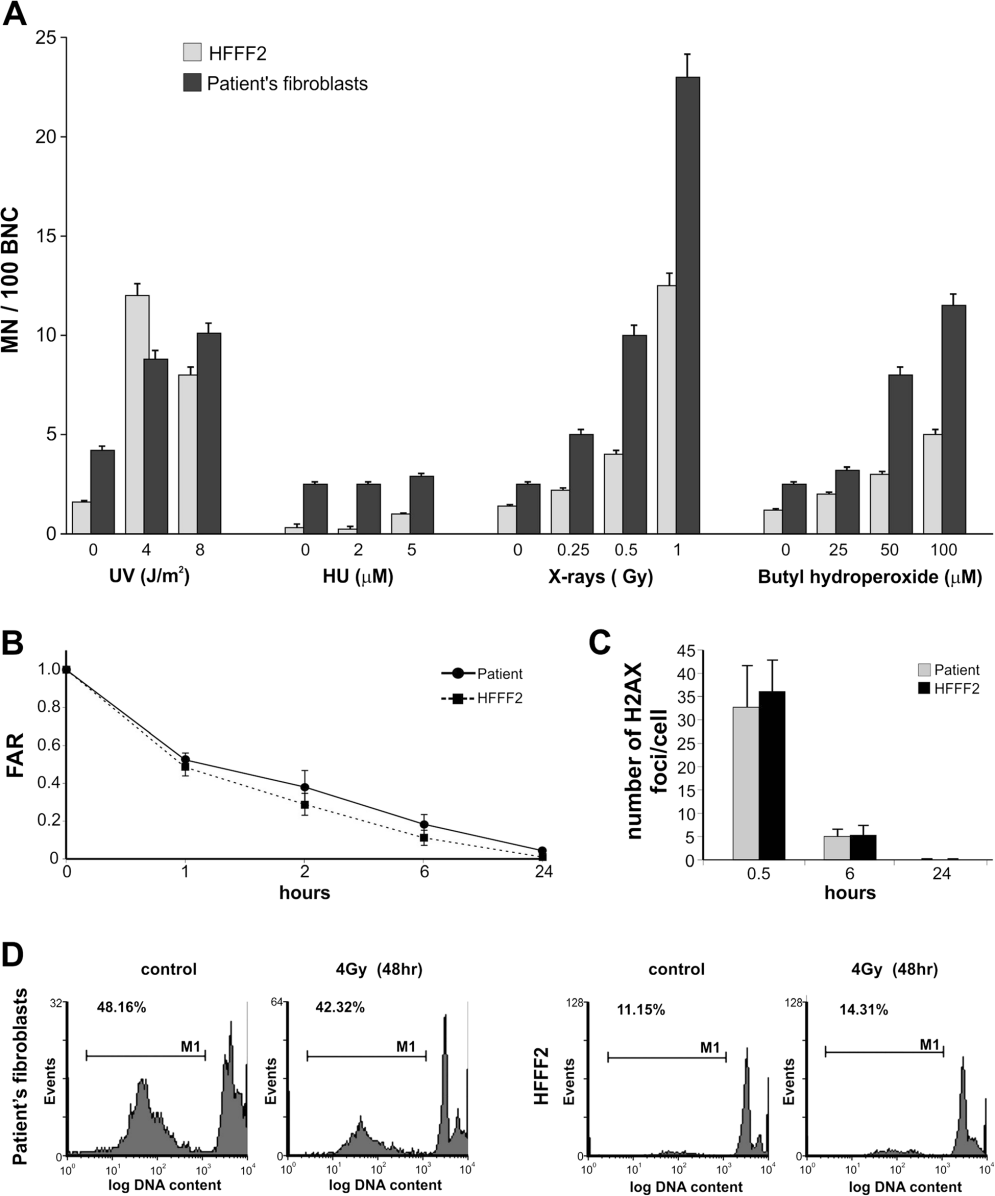
**DNA damaging analysis. (A)** MN frequency scored in binucleated cells. **(B)** Analysis of the DSBs rejoining capability of patient’s cells by PFGE after 1, 2, 6 and 24 hrs from 40 Gy X-ray treatment. **(C)** The number of phosphorylated H2AX (γ-H2AX) foci scored in cells treated with 1 Gy of X-rays and harvested 0.5, 6 and 24 hs later. **(D)** Analysis of apoptotic cells in a patient’s fibroblasts compared to HFFF2 cells.

Cells were treated with DNA damaging agents known to induce different kind of DNA lesions. No differences were scored in the frequency of MN induced by treatment with either 4–8 J/m2 of UV-C or with 2–5 μM Hydroxyurea. Contrastingly, fibroblasts established from the proband showed a higher frequency of MN after exposure to either the oxidant agent t-Butyl hydroperoxide or X-rays, indicating sensitivity towards agents able to induce DNA-strand breaks (Figure 4A).

To measure the DNA DSBs repair capability in cells established from the proband, pulsed field gel electrophoresis (PFGE) analysis was performed. Cells were irradiated with 40 Gy and the fraction of activity released (FAR) was measured 1, 2, 6 and 24 hrs after treatment. Analysis of the time-course for DSBs rejoining showed an overimposable kinetics of repair in both HFFF2 and patient’s cells (Figure 4B). In order to further characterize the repair capability of patient’s cells at biological relevant doses, a more sensitive assay was used. The number of phosphorylated H2AX (γ-H2AX) foci was scored in cells treated with 1 Gy of X-rays and harvested after 0.5, 6 and 24 hrs. Consistent with the PFGE results, the mean number of foci detected in patient’s cells was quantitatively similar to that scored in control cells (Figure 4C).

Primary fibroblasts were synchronized into G0/G1 by serum starvation for 72 hrs, allowed to resume cell proliferation in 15% FBS-containing medium, then harvested and analysed by FACS for the presence of hypodiploid DNA contents, a measure of apoptosis. Interestingly, patient’s fibroblasts showed a high percentage of apoptotic cells compared to HFFF2 cells. In particular, independently from the X-ray treatment, a percentage of apoptotic cells comprised between the 42.3% and the 48.2% was observed in patient’s fibroblasts, whereas this percentage was comprised between 11.1% and 14.3% in HFFF2 cells (Figure 4D).

## Discussion

We report on a 22-year-old patient, with malabsorption syndrome and multiple anomalies including facial dysmorphisms, growth retardation, and incomplete spina bifida, dyspraxia and presence of apoptotic bodies and lymphocytes in GI tract. The aCGH screening identified a *de novo* 150 Kb deletion in the long arm of chromosome 8 in the 8q21.2 band. Searching for the genes contained within the region of genomic imbalance, we found the *REXO1L1* gene and 3 *REXO1L2P* pseudogenes (Figure 2E). Structural variants, jointly with single-nucleotide changes, are thought to be the major contributors to genetic variation among individuals [8]. Although structural variants in some genomic regions have no obvious phenotypic consequence, others may have a significant influence in causing diseases. To interpret the clinical significance of our CNV, we used the Database of Genomic Variants containing data on hundreds of healthy individuals and publicly available databases listing pathologic chromosome aberrations [9,10]. The RP11-96G1 clone is reported to be involved in CNVs in health subjects and patients. In particular, Iafrate et al. report 9 control samples and 8 patients sharing gain/loss of genomic region covered by our clone [11]. This region is also involved in other catalogued duplications reported in the most part of structural variation studies on control samples. Only two researches specify the identified deletion in few individuals, while the genome of a single individual presents an involving of genomic region in inversion rearrangement. Often, the low CNVs frequency and the use of a single platform and technology to identify CNVs let researchers to misclassify their clinical significance. The application of aCGH technology has improved the detection of many submicroscopic chromosomal imbalances in children with dysmorphism and developmental delay/mental retardation, allowing for a specific diagnosis in patients previously considered to have an idiopathic etiology [12-14]. More recently it became clear that some of the alterations that were at first considered benign CNVs, showed to be enriched in affected population [15]. Gain/loss of genetic segments involving *REXO1L1* gene is absent from an internal database that include CNVs obtained during validation of our array CGH platform. Such validation studies used DNAs from 100 healthy control individuals. Moreover, we found only an increase of *REXO1L1* gene and *REXO1L2P* pseudogenes copy number in healthy Italian subjects by molecular techniques. On the other hand, the investigated region on chromosome 8q21.2 is resulted part of greater deletion (15 Mb at 8q21.11→q21.3) in syndromic patients [16]. Then, the *REXO1L1* gene has been identified in a genomic gain in SKBR3 breast cancer cell line [17]. Our deletion is unlikely to constitute an artefact. The confirmation of the aCGH results by molecular techniques, the absence of deletion in control samples, the overlapping with characterized genome imbalances in affected individuals, its *de novo* origin, all factors influencing the risk assessment of a CNV, corroborated opinion that our CNV is more likely to be pathogenetic in determining patient phenotype [16]. However, the potential clinical relevance of a CNV depends also on the number of genes within the imbalanced region. Our CNV is gene poor, but contains a high number of pseudogenes. Many studies consider the CNVs’ effect on contribution to protein family evolution [18]. After formation and subsequent fixation following selection or random drift, CNVs may give rise to gene duplicates or losses [19]. Many “unsuccessful” duplicates remain in the genome as pseudogenes. Protein-coding genes acting in metabolism and cellular physiological processes, that is dosage-sensitive genes, and genes putatively involved in environmental response appear significantly enriched among pseudogenes [19]. The concept of pseudogenes as “junk DNA” seems overcome. Pseudogenes often exhibit functional roles, such as gene expression, gene regulation, generation of genetic (antibody, antigenic, and other) diversity [20]. So, changes in gene and pseudogene number may contribute to development or act as susceptibility alleles of diseases [21-23]. In other cases, ‘resurrection’ of duplicated pseudogenes can result in an expressed protein [18]. Therefore, duplicated pseudogenes can be considered to be a resurrectable reservoir of diversity.

How does the deletion involving *REXO1L1* gene/*REXO1L2P* pseudogenes can influence the patient’s phenotype? The human *GOR* gene (alternative *REXO1L1* gene symbol) produces a 3′-5 exonuclease (exo) belonging to DEDDh family, composed of RNase T, RNase D and oligoribonuclease in prokaryotes, involved in 3′ maturation of several small stable RNAs, or in recycling of short oligonucleotide generated by other 3′ exo activities [24-27]. In eukaryotes there are several different DEDD superfamily 3′ exos involved in a wide range of activities including RNA maturation, nuclear mRNA surveillance and decay, and control of HBV and RNA virus infections [28-35]. Although, it was initially proposed that oligoribonuclease does not attack deoxyribonucleotides, it was recently shown that it can degrade short DNA oligos [36]. Interestingly, this highlights a possible role for oligoribonuclease in DNA repair, like its human counterpart [37]. So, we speculate that variable *REXO1L2P* pseudogene number in control population can be a protection factor against viral infections. On the contrary, our patient carrying out low copy number of the *REXO1L2P* pseudogenes could be more predisposed to contract viral illness. In fact, we also observed expression changes for a high number of heath shock genes codifying proteins (HSPs) induced by stress factors and playing a role as molecular chaperones and in antigen presentation [38].

The effects of the *REXO1L1* haploinsufficiency on the DNA damage response in patient’s cells have been investigated. First, fibroblasts established from the patient showed a higher frequency of MN after exposure to both the oxidant and ionizing radiation (IR) agents, indicating sensitivity towards agents able to induce DSBs, the most important lesions leading to chromosomal aberrations [39]. Nevertheless, a complete recovery of unrejoined DSBs after 24hs from IR in proband fibroblasts compared to control cell line and the same mean number of phosphorylated H2AX (γ-H2AX) foci detected in the patient’s cells and in control cells indicated the ability of affected fibroblasts to repair DNA damage. Both genomic instability and increased apoptosis in patient’s cells could be explained by the role of *REXO1L1* gene product. However, the exoribonuclease function of REXO1L1 protein and its involvement in the mechanism of DNA repair and apoptosis remain to be demonstrated in further experiments.

## Conclusions

In conclusion, the reported study shows a genomic disorder caused by new structural change at 8q21.2 region. The consequent gene imbalance is absent as polymorphic variants in the general population and behaves as disease determinant of the patient dysmorphic phenotype. We further illustrated that affected fibroblasts share genomic instability and alteration of mRNA expression profile. Consequently, our results support the hypothesis that low copy gene number within *REXO1L1* cluster identified in the patient could play a significant role in his clinical and cellular phenotype. If this genomic imbalance is associated to a major susceptibility to viral infections, that could explain both the high apoptosis level and the high number of differentially expressed heath shock genes, is currently under investigation.

Our contention is the identification of low size chromosomal aberrations by standard karyotyping methods is difficult contributing to its rarity in the recognition of new microdeletion syndromes. However, the use of aCGH screening in dysmorphic patients with global developmental delay, progeroid signs and gastrointestinal anomalies should increase the recognition of individuals carrying this novel deletion.

## Consent

Informed consent was obtained prior to initiating our investigation.. Written informed consent was obtained from the patient for publication of this case report and any accompanying images. A copy of the written consent is available for review by the Editor of this journal. Permission to publish the patient’s photo was not granted.
